# Growth physiology, genomics, and proteomics of *Desulfurivibrio dismutans* sp. nov., an obligately chemolithoautotrophic, sulfur disproportionating and ammonifying haloalkaliphile from soda lakes

**DOI:** 10.3389/fmicb.2025.1590477

**Published:** 2025-05-23

**Authors:** Dimitry Y. Sorokin, Alexander Y. Merkel, Rustam H. Ziganshin, Ilya V. Kublanov

**Affiliations:** ^1^Winogradsky Institute of Microbiology, Federal Research Centre of Biotechnology, Russian Academy of Sciences, Moscow, Russia; ^2^Department of Biotechnology, Delft University of Technology, Delft, Netherlands; ^3^Shemyakin-Ovchinnikov Institute of Bioorganic Chemistry, Russian Academy of Sciences, Moscow, Russia; ^4^Department of Plant Pathology and Microbiology, The Robert H. Smith Faculty of Agriculture, Food and Environment, The Hebrew University of Jerusalem, Rehovot, Israel

**Keywords:** extremophiles, alkaliphiles, soda lakes, sulfur disproportionation, reversed sulfate reduction, nitrate reduction, proteomics

## Abstract

Elemental sulfur disproportionation combined with obligate autotrophy is a unique type of sulfur-based anaerobic metabolism known in a limited number of bacteria, primarily found among the members of *Desulfobacterota* phylum. Until recently, the only characterized alkaliphilic representative of this group was *Desulfurivibrio alkaliphilus*, originally isolated as an H_2_-dependent sulfur reducer. In this study, we describe the properties of a novel species within this genus, *Desulfurivibrio dismutans* strain AMeS2, which was originally enriched and isolated from a soda lake sample as an autotrophic elemental sulfur disproportionating bacterium. Similar to *D. alkaliphilus* AHT 2^T^, *D. dismutans* AMeS2 is an obligately alkaliphilic and moderately salt-tolerant autotrophic bacterium. In contrast to known neutrophilic sulfur disproportionating bacteria, it is capable of disproportionating sulfur without Fe(III). It can also grow by dissimilatory sulfur reduction to sulfide or nitrate reduction to ammonium (DNRA) with formate (but not with H_2_) as the electron donor. The addition of formate to sulfur-disproportionating AMeS2 culture significantly increased the sulfur-reducing activity but did not completely abolish the oxidative branch of sulfur disproportionation. Genome analysis confirmed the presence of dissimilatory sulfur oxidation and dissimilatory sulfur and nitrate reduction machineries in the strain. S^0^ disproportionation occurs by means of cytoplasmic dissimilatory sulfite reductase (Dsr) donating electrons to, and periplasmic polysulfide reductase (PsrABC) receiving electrons from the menaquinone pool. Nitrate reduction to ammonium (DNRA) occurs by the combined action of a membrane formate dehydrogenase FdnGHI, periplasmic nitrate reductase, and octaheme *c* ammonifying nitrite reductase. Autotrophic growth is enabled by the Wood–Ljungdahl pathway (WLP). The genome also encodes proteins that presumably connect the oxidative branch of sulfur disproportionation with the carbon (WLP) cycle. Comparative proteomics of cells grown by sulfur disproportionation and formate-dependent DNRA demonstrated overexpression of the genes encoding Psr and rDSR at sulfur-disproportionating conditions, confirming their key role in this process. On the contrary, the genes encoding DNRA proteins are upregulated in the presence of nitrate. Thus, genomic and proteomic analyses revealed the pathways for energy conservation in a new representative of *Desulfurivibrio* growing at DNRA and under the thermodynamically challenging conditions of sulfur disproportionation.

## Introduction

Due to the high number of redox states from the most reduced sulfide (−2) until the most oxidized sulfate (+6), and its overall abundance, sulfur compounds participate in many important dissimilatory redox reactions driven by diverse prokaryotes. Among the sulfur compounds with intermediate valence, elemental sulfur in the form of an S_8_ ring is the most inert due to its extremely low water solubility. Despite that, it can be used as both an electron acceptor and an electron donor by a wide variety of sulfur-respiring anaerobes and sulfur-oxidizing prokaryotes, respectively (Crane, 2019; Ghosh and Dam, 2009; Wasmund et al., 2017; Zhou et al., 2025). In addition to the abovementioned unidirectional redox transformations with external electron donors or acceptors, elemental sulfur can be disproportionated (i.e., dismutated or fermented) when one of its parts is oxidized to sulfate. In contrast, another part is reduced to sulfide. In addition to elemental sulfur, disproportionation of mixed valence thiosulfate (S^2−^ + S^4+^) and sulfite (S^4+^) is also known for the members of the bacteria domain (Bak and Cypionka, 1987; Finster, 2008; Slobodkin and Slobodkina, 2019).

While many species of sulfate-reducing bacteria quite commonly ferment soluble thiosulfate and sulfite, disproportionation of insoluble elemental sulfur is a thermodynamically challenging process known for a minimal range of anaerobic bacteria, the majority of which are obligate autotrophs (Slobodkin and Slobodkina, 2019; Wasmund et al., 2017). The majority of the neutrophilic sulfur disproportionators can do it only in the presence of Fe(III) compounds, such as ferrihydrite, which are involved in sulfur oxidation and bind sulfide in the form of insoluble FeS. This makes the dismutation reaction exergonic enough to support weak growth of these bacteria. In contrast, at high pH microbial sulfur disproportionation proceeds without adding ferrihydrite, probably because the produced sulfide chemically interacts with remaining sulfur to form polysulfides (S_n_^2−^), which are stable in anoxic alkaline solutions. So far, this process has been demonstrated for a single natronophilic bacterium, *Desufurivibrio alkaliphilus*, from the *Desulfobulbales* order (Poser et al., 2013; Thorup et al., 2017; Sorokin and Merkel, 2020), isolated from anoxic sediments of Egyptian soda lakes as an H_2−_ dependent sulfur reducer (Sorokin et al., 2008). The genome/transcriptome analysis of *D. alkaliphilus* has shown the typical array of proteins characteristic of neutrophilic *Desulfobacterota* sulfur disproportionators, including PsrABC for the reductive branch and Dsr-Apr complexes for the oxidative branch of sulfur disproportionation and the Wood–Ljungdahl pathway (WLP) for autotrophic carbon fixation (Melton et al., 2016; Thorup et al., 2017). Apart from sulfur disproportionation, *D. alkaliphilus* can grow by H_2−_ and sulfide-dependent dissimilatory nitrate reduction to ammonia (DNRA). For the latter, *D. alkaliphilus* uses sulfide quinone oxidoreductase (Sqr), which oxidizes sulfide to polysulfide, and a combination of periplasmic dissimilatory nitrate reductase NapAGHD and a non-canonical ammonifying octaheme *c* nitrite reductase homologous to a recently characterized enzyme from *Trichlorobacter ammonificans* (Sorokin et al., 2023). Although it was shown that the grown cells of sulfate-reducing bacteria can oxidize sulfide in presence of nitrate, this pair of donor and acceptor did not support their growth (Haveman et al., 2004). Therefore, *D. alkaliphilus* is the only bacterium known to be capable of growth by this type of respiration.

It was recently reported that uncultured *D. alkaliphilus* relatives (the members of the same genus and family) inhabit various anoxic environments, such as Siberian soda lake sediments (Vavourakis et al., 2019) as well as pH circumneutral deep terrestrial subsurface (Bell et al., 2022) environments and Sb-mining tails (Sun et al., 2022). There are also two reports about *Desulfurivibrio* interacting with electrodes using *e*-pili similar to those of *Geobacter* species (Walker et al., 2018). They are capable of forming particularly dense biofilms on the anode surface (Ni et al., 2019; de Rink et al., 2022; Izadi and Schröder, 2022; Mickol et al., 2021; Shen et al., 2023), implicating their tendency to exchange electrons with insoluble acceptors.

This paper presents results of physiological, genomic, and proteomic characterization of strain AMeS2, representing a second species in the genus *Desulfurivibrio* enriched from anoxic sediments of soda lakes in southwestern Siberia, for which we suggest the name *D. dismutans*. It is a haloalkaliphilic anaerobic chemoilithoautotroph capable of growth either by elemental sulfur disproportionation or by formate-dependent DNRA.

## Materials and methods

### Enrichment conditions and isolation of pure culture

Mix sulfidic sediments from five soda lakes sampled in 2011 in Kulunda Steppe (Altai, Russia) with total salt concentration of brines 90–110 g L^−1^, pH 10.1–10.3, and soluble carbonate alkalinity 1.1–1.7 M were used as inoculum to enrich for haloalkaliphilic sulfur-disproportionating bacteria. The enrichment medium was based on sodium carbonate–bicarbonate buffer with pH 10 (after heat sterilization) and contained 0.5 M total Na^+^ as sodium carbonates and 0.1 M NaCl. NH_4_Cl (2–4 mM) served as N-source and was added after sterilization together with 1 mM MgCl_2_, 1 mL L^−1^ each of acidic trace metals and vitamins (Pfennig and Lippert, 1966), and 1 mL L^−1^ of alkaline sodium tungstate/selenate solution (Plugge, 2005). Notably, 40 mL medium portions were added to 50 mL serum bottles supplied with 0.4 g of crystalline sulfur (SigmaAldrich, USA), washed multiple times with distilled water, and sterilized at 110°C for 20 min. The bottles were inoculated with 2 mL of fine sediment suspension obtained after 30 min of gravity sedimentation of a heavy sandy fraction from a 1:2 sediment brine mixture. After sealing with sterile butyl rubber stoppers, the medium was made anoxic by applying first “cold boiling” under vacuum to remove dissolved air, followed by three cycles of flushing with sterile argon gas. Final reduction was achieved by adding 0.2 mM Na_2_S by syringe from a 1 M anoxic stock solution. The bottles were incubated at 28°C in the dark, and the development was followed by the formation of yellow-colored polysulfide and measuring total sulfane formation (sulfide + polysulfide sulfane) compared to an uninoculated control. When sulfane reached a 10 mM level, one of the positive enrichments was subcultured 3 times at 1:100 dilution to obtain a sediment-free culture, then subjected to serial dilutions in 10 mL volume in flat-bottom 20 mL serum bottles, and the last positive dilution of (−9) was used to attempt soft shake agar colony formation. This appears to be difficult, considering the problem with the even distribution of sulfur particles. However, probably due to the formation of soluble polysulfide, colony formation was achieved after 1 month of incubation in up to (−6) dilution (Supplementary Figure S1). It resulted in pure culture isolation capable of growth in the original liquid mineral medium with the only substrate of elemental sulfur. The strain was designated AMeS2.

### Genome sequencing and analysis

Genomic DNA was isolated from AMeS2 cells grown with sulfur and formate using the FastDNA™ SPIN Kit for Soil (MP Biomedicals, USA). A shotgun metagenome library was prepared using the KAPA HyperPlus Library Preparation Kit (KAPA Biosystems, UK) according to the manufacturer’s protocol, and sequencing was carried out on the NovaSeq 6,000 system (Illumina, San Diego, CA, USA) using reagents, allowing sequencing 100 nucleotides from each end of the read. The genome was assembled using Unicycler v0.5.0 (Wick et al., 2017), deposited in the GenBank under accession number GCA_029210385.1, and annotated using PGAP (Tatusova et al., 2016). The locus tag designations of the respective RefSeq annotation (GCF_029210385) are P0N66_RSXXXXX (where X are numbers), but in the article, they are abbreviated to RSXXXXX.

To infer phylogeny of the strain, 120 single-copy conserved bacterial marker proteins were used according to the Genome Taxonomy Database (GTDB) (Rinke et al., 2021) and aligned using GTDB-Tk software kit (Chaumeil et al., 2019). The trees were built with the IQ-TREE2 program (Minh et al., 2020) with fast model selection via ModelFinder (Kalyaanamoorthy et al., 2017), ultrafast bootstrap approximation (Minh et al., 2013), and an approximate likelihood-ratio test for branches (Anisimova and Gascuel, 2006). Average Nucleotide Identity (ANI) was calculated using Pyani 0.2.12 (Pritchard et al., 2016); Average Amino acid Identity (AAI) was estimated by the EzAAI v1.1 (Kim et al., 2021), and DNA–DNA hybridization (DDH) by the Genome-to-Genome Distance Calculator 3.0 online tool.^1^

Manual refining the Prokaryotic Genome Annotation Pipeline (PGAP) autoannotation was performed similarly to the pipelines proposed by Toshchakov et al. (2015). The following software packages and tools were used: Geneious Prime 2019, TMHMM 2.0 (Krogh et al., 2001); SignalP 6.0 (Teufel et al., 2022); hmmscan (Potter et al., 2018). VolcanoPlot was inferred in RStudio (R version 4.4.2, packages *ggplot2*, *dplyr*, and *ggrepel*). A metabolic map of sulfur disproportionation was drawn in CorelDRAW x5.

### Phenotypic characteristics and growth experiments

Cell morphology and tests of various combinations of electron donors/acceptors were studied using the aforementioned standard mineral medium at 0.6 M total Na^+^ and pH 10. Life cell morphology was observed using a phase contrast microscope (Zeiss Axioplan Imaging 2, Germany). For electron microscopy (JEM-100 JEOL instrument, Japan), cells were centrifuged, resuspended in 0.5 M NaCl, and fixed with 4% paraformaldehyde at 4°C for 2 h. Fixed cells were centrifuged again, resuspended in 0.5 M NaCl, and positively stained with 1% uranyl acetate for total microscopy or postfixed with 1% (final) OsO_4_ for thin-section electron microscopy. The sections were stained with 1% lead citrate and uranyl acetate.

Substrate tests as well as the verification of dependence on vitamins and yeast extract (0.1 g L^−1^) as a source of the growth factors were performed in 23 mL serum bottles with 10 mL cultures (in case of H_2_ and CO—with 5 mL) in triplicates using uninoculated medium without test substrate or factor as the control. For growth dynamics (sulfur disproportionating and DNRA), 200 mL cultures were incubated in 300 mL screwcap bottles with butyl rubber stoppers. Apart from sulfur, polysulfide (average formula S_5_^2−^), thiosulfate and sulfite (10 mM) were tested as substrates for disproportionating growth, and sulfur, thiosulfate, sulfite, and sulfate—as the electron acceptors with either H_2_ (20% in the gas phase) or formate (50 mM) as electron donors. Other tested electron acceptors with the mentioned donors included nitrate (10 mM), nitrite 5 mM, arsenate, fumarate (5 mM each), and ferrihydrite (20 mM). Acetate, pyruvate, lactate, succinate (5 mM), and yeast extract (0.5 g L^−1^) were tested as the carbon and energy source with sulfur as the electron acceptor. The influence of total Na^+^ (at pH 10) and pH (at total Na^+^ 0.6 M) was investigated in growing culture and on the level of sulfidogenic activity of resting cells grown under disproportionating conditions. For the pH ranging 6.5–8, a combination of HEPES and potassium phosphate buffers (both at 50 mM) was used with NaCl as the main salt; NaHCO_3_ served as the primary buffer and salt for the pH ranging 8–9 adjusted either with CO_2_ in the gas phase or Na_2_CO_3_; the pH range above 9 was established using sodium bicarbonate–carbonate mixture. The final pH at the end of the experiments was considered the actual pH.

### Chemical analyses

Cell growth was monitored by OD600 (in case of sulfur-containing media, after 30-s centrifugation at 2,000*g* in 2 mL Eppendorf tubes). The concentration of cellular protein in cell suspension experiments was measured using the Lowry method. Prior to measurements, the pellets were washed with 0.5 M NaCl solution containing 50 mM HCl to remove traces of residual sulfane. A sum of free HS^−^ and reduced (sulfane) atoms of polysulfide was measured (as total sulfane) by the methylene blue method (Trueper and Schlegel, 1964) after fixing supernatants in 10% Zn acetate. For sulfate analysis, the supernatants were acidified with 2 M HCl to pH 3, purged with N_2_ to remove sulfide, and centrifuged to remove sulfur formed by acid hydrolysis of polysulfides. Sulfate concentration was measured by non-suppressed anion high-performance liquid chromatography (HPLC) (Eppendorf-Netheler-Hinz GmbH, Division Biotronic, Germany) with a BT11AN column and a refractometer detector. Prior to measurements, the samples were neutralized and diluted in Milli-Q. A solution containing 1 mM Na_2_CO_3_ and 1.2 mM NaHCO_3_ was used as eluent with a flow rate of 1.5 mL min^−1^. Internal zero-valent sulfur content of polysulfides was determined after acidic hydrolysis followed by acetone extraction, cyanolysis, and spectrophotometric detection of Fe(CNS)_3_ at 460 nm according to Sorbo (1957). Nitrate, nitrite, and ammonia were analyzed spectrophotometrically at 420, 540, and 623 nm, respectively, as described previously (Sorokin et al., 2023).

### Proteomic analysis

To investigate the expression of the genes of key proteins determining two different modes of respiration of AMeS2, its cells were grown at optimal salt and pH conditions via sulfur disproportionating and formate-dependent nitrate reduction to ammonium for three consecutive transfers. The cells from the last transfer (200 mL of cultures under each condition) were collected during the late exponential growth phase by centrifugation, washed once in 0.6 M NaCl, and the pellets were kept at −20°C until further processing. Prior to centrifugation, the excessive amount of unused sulfur was removed by gravity separation.

Cell lysis was done as follows: cell pellets were resuspended in 500 μL of reduction and alkylation buffer [4% sodium deoxycholate (SDC), 100 mM TRIS pH 8.5, 10 mM tris(2-carboxyethyl)phosphine hydrochloride (TCEP), 40 mM 2-chloroacetamide (2-CAA)] and sonicated for 20 s using ultrasonic cell disrupter VirSonic 100 (VirTis, SP Industries Inc. USA). The solution was boiled for 10 min and centrifuged at 15,000*g* for 15 min after cooling to room temperature. Protein concentration in cell lysates was determined using a Bradford reagent. An aliquot of cell lysate (100 μg of protein) was diluted eight times with 100 mM TRIS pH 8.5, and 1 μg of sequencing-grade modified trypsin (Promega, Madison, WI, USA) was added. Digestion was performed at 37°C overnight. Peptides were desalted using Empore membrane solid phase extraction (SPE, CDS Analytical Inc, USA) disks (styrenedivinylbenzene–reverse phase sulfonate [SDB-RPS]) StageTips as described previously (Kulak et al., 2014) with minor modifications: peptides were acidified to a final concentration of 0.1% trifluoroacetic acid (TFA), and 20 μg was loaded on three 14-gauge StageTips plugs. Ethylacetate/1% TFA (125 μL) was added, and the StageTips were centrifuged at 1,000*g*. After washing the StageTips using two wash steps of 100-μL ethylacetate/1% TFA and one of 100-μL 0.2% TFA consecutively, peptides were eluted by 60 μL of elution buffer (80% acetonitrile, 5% ammonia). The collected material was dried completely using a SpeedVac centrifuge (Savant, SpeedVac concentrator, Thermo Fisher Scientific) and stored at −80°C before analyses.

Peptides were dissolved in loading buffer (2% ACN, 0.1% TFA) and sonicated for 1 min in an ultrasonic water bath (Elma, Elmasonic S100, Elma Schmidbauer GmbH, Germany) before nano-flow liquid chromatography tandem mass spectrometry (nano-flow LC–MS)/MS analysis. Approximately 1 μg of peptides were loaded to a home-made trap column 50 mm × 0.1 mm, packed with Reprosil-Pur 200 C18-AQ 5 μm (Dr. Maisch) sorbent, in the loading buffer (2% ACN, 98% H_2_O, 0.1% TFA) at 4.2-μL/min flow and separated at RT in a home-packed fused-silica column 300 mm × 0.1 mm packed with Reprosil-Pur 120 C18-AQ 1.9 μm (Dr. Maisch) into an emitter prepared with P2000 Laser Puller (Sutter, USA). Reverse-phase chromatography was performed with an Ultimate 3,000 Nano LC System (Thermo Fisher Scientific), which was coupled to the Q Exactive HF mass spectrometer (Thermo Fisher Scientific) via a nanoelectrospray source (Thermo Fisher Scientific). Peptides were loaded in buffer A (0.1% (v/v) formic acid) and eluted with a linear 120 min gradient of 4–55% buffer B (0.1% (v/v) formic acid, 80% (v/v) acetonitrile) at a flow rate of 500 nL/min. After each gradient, the column was washed with 95% buffer B for 5 min and re-equilibrated with buffer A for 5 min. MS data were acquired with an automatic switch between a full scan and up to 10 data-dependent MS/MS scans (topN method). Target value for the full scan MS spectra was 3 × 10^6^ in the 320–1,600 *m/z* range with a maximum injection time of 35 ms and a resolution of 70,000. Precursors were isolated with a 1.4 *m/z* window and a fixed first mass of 100.0 *m/z*. Precursors were fragmented by higher-energy C-trap dissociation (HCD) with a collision energy of 28%. MS/MS scans were acquired at a resolution of 17,500 with an ion target value of 1 × 10^5^ with a maximum injection time of 50 ms. Repeat sequencing of peptides was minimized by excluding the selected peptide candidates for 30 s.

MS raw files were analyzed by MaxQuant software version 1.5.6.5 (Cox and Mann, 2008), and peptide lists were searched against the *D. dismutans* translated genome and a common contaminants database by the Andromeda search engine (Cox and Mann, 2011) with cysteine carbamidomethylation as a fixed modification and N-terminal acetylation and methionine oxidations as variable modifications. The false discovery rate was set to 0.01 for proteins and peptides with a minimum length of seven amino acids and was determined by searching a reverse database. Enzyme specificity was set to trypsin, and a maximum of two missed cleavages was allowed in the database search. Peptide identification was performed with an allowed initial precursor mass deviation up to 10 ppm and an allowed fragment mass deviation of 0.02 Da. Proteins matching the reversed database were filtered out.

Comparative label-free protein quantification was made using MaxLFQ approach (Cox et al., 2014), and estimation of the mole fraction of each identified protein within the proteome was made using riBAQ algorithm (Shin et al., 2013).

## Results and discussion

### General genome properties and phylogenetic position of strain AMeS2

The genome sequence was assembled into 33 contigs with N50 and L50 of 330 kb and 4 kb, respectively. The total length of the genome was 2.8 Mb with a GC content of 60%. Among 2,553 predicted genes in the RefSeq annotation (GCF_029210385.1) 2,491 were protein-coding sequences.

**Figure 1:**
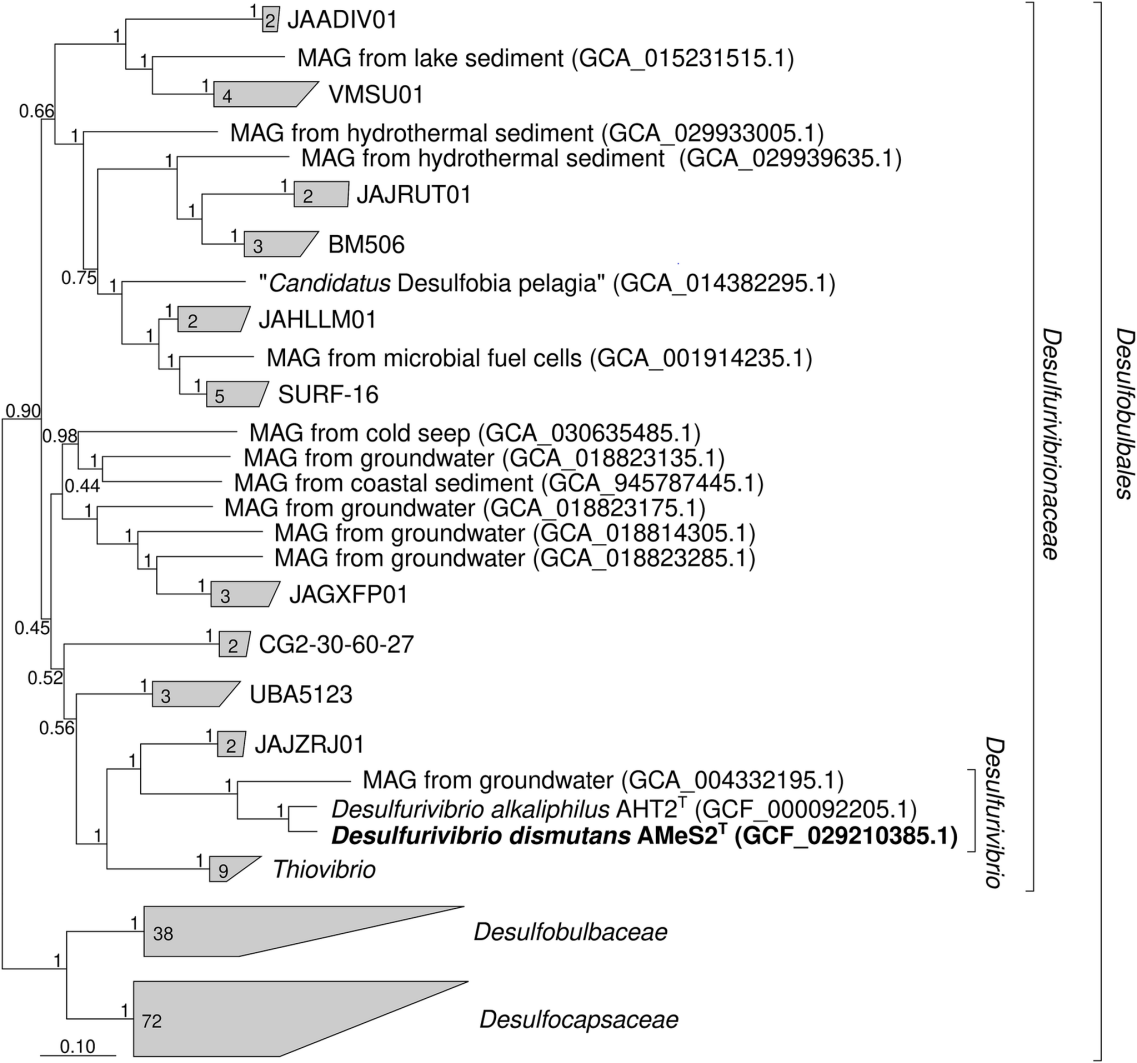
Phylogenetic position of *Desulfurivibrio dismutans* AMeS2^T^ (in bold) based on sequence analyses of a concatenated alignment of 120 single-copy conserved bacterial protein markers (names of uncultured clusters are given according to the Genome Taxonomy Database Release 09-RS220) (Parks et al., 2020). The trees were built using the IQ-TREE 2 program (Minh et al., 2020) with ultrafast bootstrap approximation (Minh et al., 2013) as well as an approximate likelihood-ratio test for branches (Anisimova and Gascuel, 2006). The bootstrap consensus tree is shown with values placed at the nodes. Bar, 0.10 changes per position.

*Desulfurivibrio alkaliphilus* AHT 2^T^ was the most closely related to the AMeS2, with the validly published name: 16S ribosomal RNA (rRNA) gene sequence identity between them was 97.8%. Phylogenomic analysis based on a 120-protein concatenated sequence confirmed the placement of AMeS2 within the *Desulfurivibrio* genus of the family *Desulfurivibrionacea*, class *Desulfobulbia* (Figure 1). The ANI, AAI, and DDH values between AMeS2 and *D. alkaliphilus* AHT 2^T^ were 86.84, 84.93, and 26.60%, respectively, all of which are significantly below the species delineation (Meier-Kolthoff et al., 2013), indicating that AMeS2 is a representative of a new species of the genus for which the name *D. dismutans* is proposed. Besides AMeS2 and *D. alkaliphilus* AHT 2^T^ *Desulfurivibrionacea* contained several metagenome-assembled genomes (MAGs) from various habitats, as well as *Thiovibrio frasassiensis*, which is also capable of autotrophic sulfur disproportionation (Aronson et al., 2023). Our phylogenetic analysis and GTDB classification agree in placing the *Thiovibrio* genus within the family *Desulfurivibrionacea*, rather than representing a separate family as originally proposed by Aronson et al. (2023).

### Morphological features

**Figure 2:**
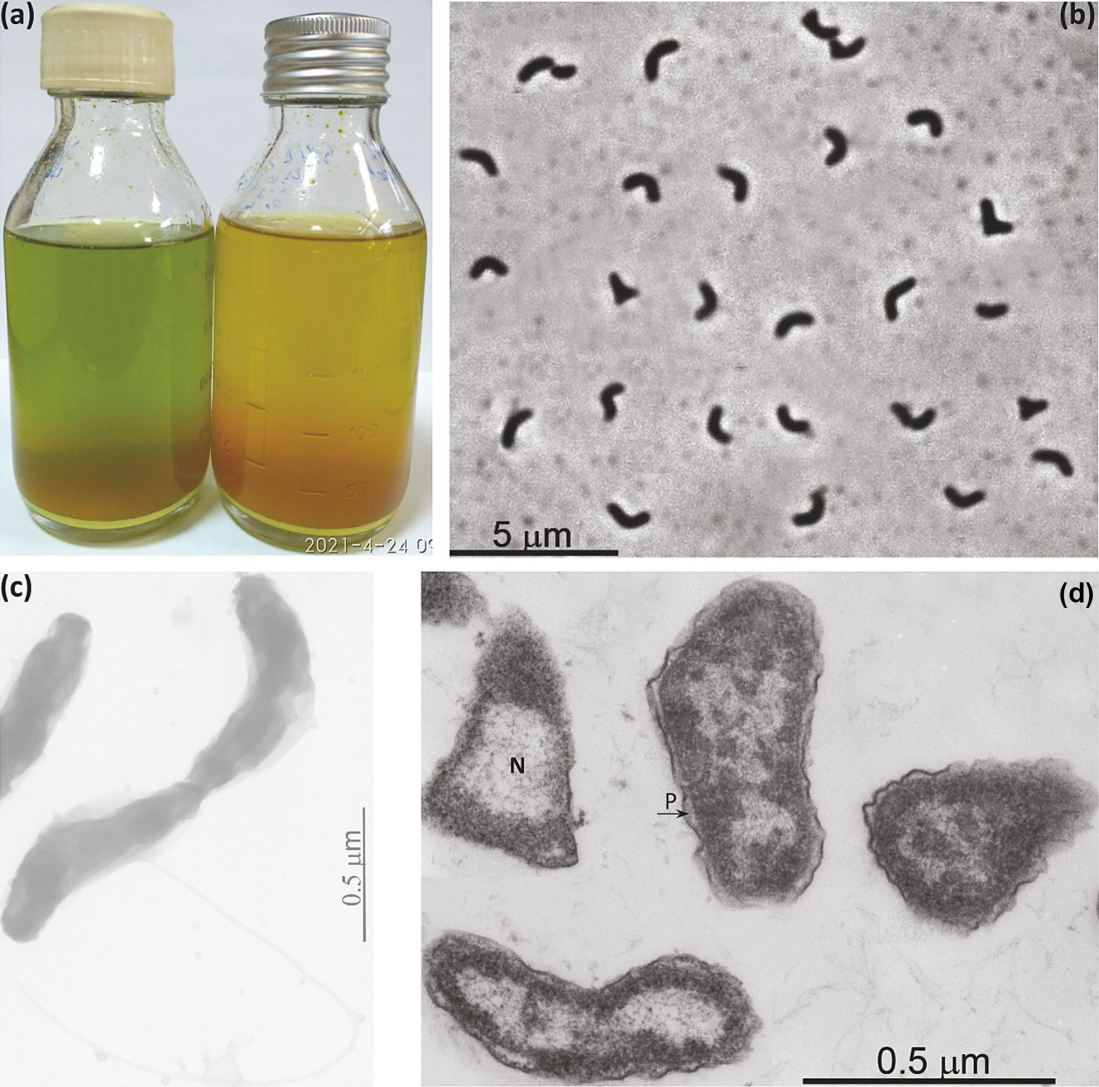
Macro- **(a)** and micro- **(b–d)** morphology of strain AMeS2 grown at 0.6 M total Na^+^ and pH 10. **(a)** Difference in polysulfide formation between sulfur disproportionating (left, with domination of S_3_^2−^) and in sulfur + formate culture (right, with domination of S_5_^2−^); **(b)** Phase contrast microphotograph of cells in sufur-disproportionating culture; **(c)** Total electron microscopy showing flagellation; **(d)** Thin section electron microscopy showing large nucleoid region (N) and periplasmic compartment (P).

Similar to *D. alkaliphilus*, cells of AMeS2 are small motile vibrios; however, their size varies significantly depending on the growth conditions. They have 1–2 thick subpolar flagella but no obvious other types of appendages, such as pili or fimbria, and have ultrastructural organization typical for Gram-negative bacteria (Figures 2b–d). During its growth on liquid medium with elemental sulfur alone, the color of the medium turned green. It remained so until late logarithmic phase due to the domination of a short-length trisulfide (S_3_^2−^). In contrast, cultures supplemented with formate rapidly turned orange, accumulating longer chain polysulfides (average as S_5_^2−^) (Figure 2a).

### Growth physiology and activity tests with resting cells

Experiments with various electron donors and acceptors showed a very narrow metabolic potential of AMeS2 limited to anaerobic chemolithoautotrophic growth by elemental sulfur disproportionation (Equation 1) and formate-dependent DNRA (Equation 2). In contrast to *D. alkaliphilius*, AMeS2 was incapable of utilizing H_2_ and failed to grow by sulfide-dependent DNRA. External vitamins and yeast extract as a source of growth factors were not essential and did not stimulate the growth; therefore, further growth experiments were performed without them.


(1) $${\mathrm{4S}}^{0}+{\mathrm{4H}}_{2}O\to {{\mathrm{SO}}_{4}}^{2-}+{\mathrm{3HS}}^{-}+{\mathrm{5H}}^{+}$$

(2) $${\mathrm{4HCOO}}^{-}+{{\mathrm{NO}}_{3}}^{-}+{\mathrm{2H}}_{2}O\to {{\mathrm{4HCO}}_{3}}^{-}+{\mathrm{NH}}_{3}+{\mathrm{OH}}^{-}$$

**Figure 3:**
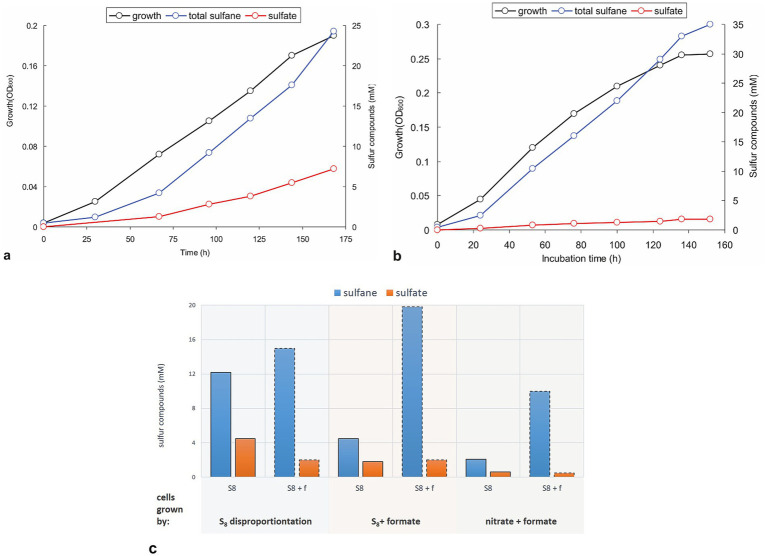
Growth dynamics and activity of resting cells of strain AMeS2 grown under different conditions. All incubations were performed in carbonate/bicarbonate buffer at pH 10 and 0.6 M total Na^+^. **(a,b)** Autotrophic growth and sulfur products formation at sulfur disproportionating conditions or on sulfur + 50 mM formate, respectively; **(c)** Comparison of the rates of S^0^ disproportionation (S_8_; bars with solid outline) and formate oxidation by S^0^ (S_8_ + f; bars with dashed outline) in AMeS2 resting cells (cell protein = 0.4 mg mL^−1^) pregrown at three different conditions: sulfur disproportionating, sulfur + 50 mM formate and 10 mM nitrate + 50 mM formate. The rates are shown as the amount of reduced (as total sulfanes; blue bars) and oxidized (sulfate; orange bars) sulfur products formed by the resting cells, incubated under each condition (S_8_ or S_8_ + f). The results are the mean of three replicate experiments for cultures and two for cell suspensions.

Growth dynamics of sulfur-disproportionating cultures are shown in Figure 3a. As aforementioned, the strain’s growth was accompanied by the formation of short-length polysulfides (greenish) which turned yellowish-orange at the later growth phases, whereby the biomass growth had a rather linear than exponential trend. After 7 days of incubation, the ratio of total reduced sulfane (sum of free sulfide and polysulfide sulfane) to sulfate was 3.3:1.0, a little above the theoretical 3.0:1.0 (Equation 1). Adding formate as another electron donor slightly increases the growth rate and the biomass yield of the AMeS2, with a much more pronounced effect on the (reduced):(oxidized) sulfur products ratio. Although the cultures did not completely abolish disproportionation, the sulfur catabolism shifted mainly to the reductive mode with a (sulfane):(sulfate) ratio varied from 8.3:1.0 to 19.3:1.0 in the initial and the late growth phases, respectively (Figure 3b). This formate-enforced shift in sulfur catabolism was further confirmed in experiments with resting cells obtained from sulfur-disproportionating and formate + sulfur-grown cultures (Figure 3c). Moreover, cells grown in sulfur-independent mode (formate + nitrate, see below) exhibited significant suppression of the sulfur-oxidation branch. In comparison, the sulfur-reducing branch was still active (Figure 3c). There was a visible difference in color of the AMeS2 biomass grown in three different modes: the sulfur-disproportionating cell mass was slightly greenish, the formate + sulfur-grown cells—slightly pinkish—and the DNRA cells—bright red (Supplementary Figure S2). The greenish color of disproportionating cells might result from the high concentration of sirohemes from DsrAB. In contrast, its concentration in cells grown with formate + sulfur should be much lower. The bright pink color of the DNRA cells is related to the high concentration of the octaheme *c* containing ammonifying nitrite reductase (see below).

An important question that until now was still unclear for alkaliphilic sulfur disproportionators is whether these organisms are capable of active polysulfide catabolism or if it is an end product of the chemical reaction of synthesized sulfide in excess of sulfur. Indeed, short-length polysulfides (trisulfides with an average formula S_3_^2−^) are an intermediate of sulfur catabolism, and both the AMeS2 culture growing on S^0^ and the resting cells pre-grown on S^0^ can metabolize long-chain polysulphides with an average formula S_5_^2−^. However, growth tests with different starting polysulfide concentrations in the absence of S^0^ were negative. These observations support the idea that the long-chain polysulfides are the end products of sulfur reduction. Although polysulfides are less toxic than the free sulfide, they might accumulate at growth-inhibiting concentrations. Since the resting cells can still metabolize polysulfides, the inability of AMeS2 to grow on this substrate is most probably related to thermodynamic constraints, particularly the oxidative branch of S^0^ disproportionation, or challenges in importing long-chain sulfur molecules into the cytoplasm (Poser et al., 2013; Amend and LaRowe, 2019).

**Figure 4:**
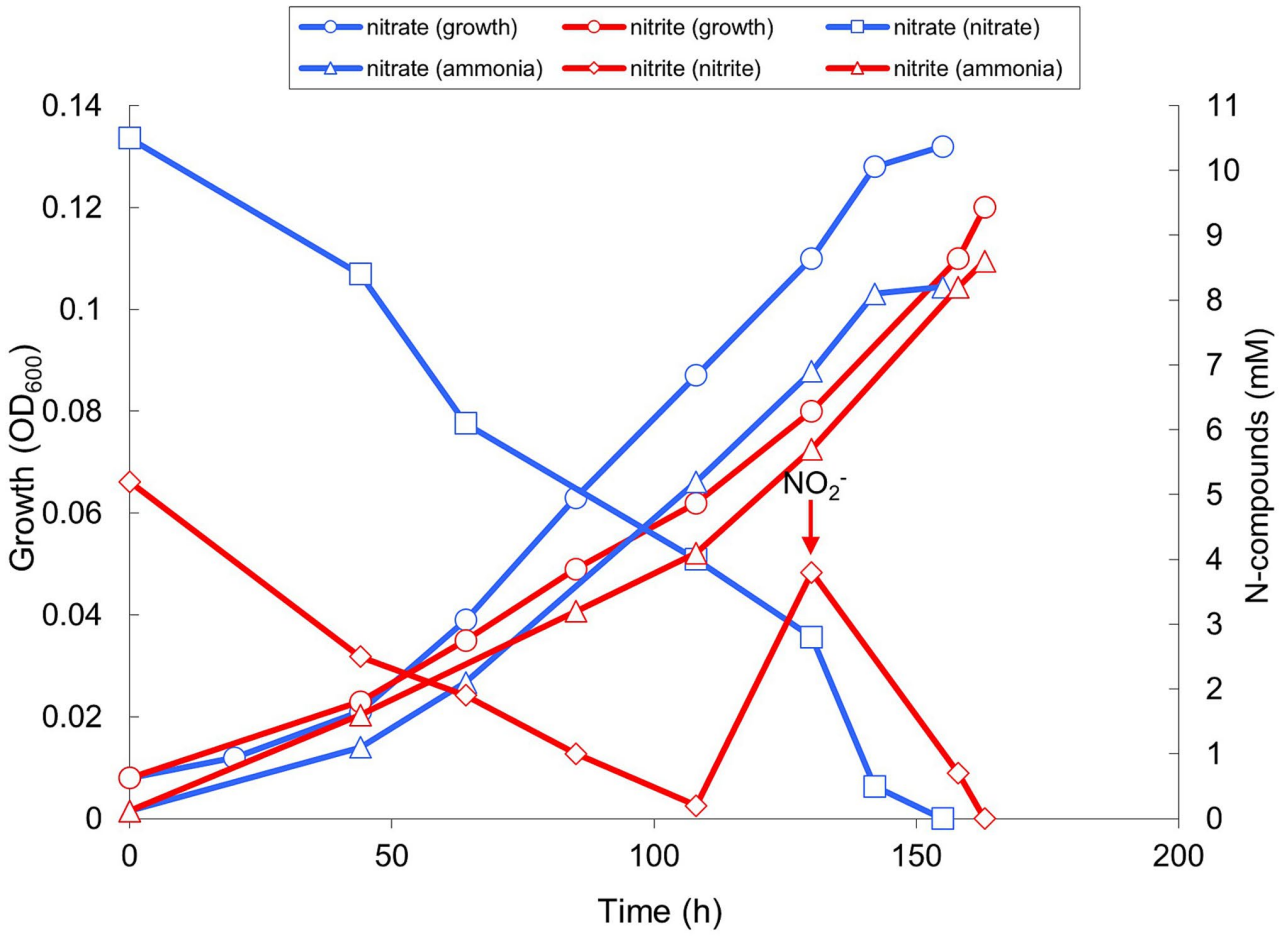
Growth and product formation of strain AMeS2 in ammonifying conditions (at pH 9.5 and 0.6 M total Na^+^) with 50 mM formate as the electron donor with either 10 mM nitrate or 2 mM × 5 mM nitrite as the electron acceptors. The medium was reduced with 0.2 mM sulfide. The arrow indicates a second addition of 5 mM nitrite. The initially added ammonium (2 mM) was subtracted from the measured values. The results are the mean of three replicate experiments with nitrate and two with nitrite.

Apart from sulfur-based metabolism, AMeS2 grew by ammonifying nitrate/nitrite respiration with formate but not hydrogen as the electron donor. With both acceptors, the growth was only possible at low redox potential achieved by medium reduction with sulfide (but not cysteine), albeit sulfide could not replace formate as the electron donor. As opposed to nitrate added at concentrations of 10 mM, nitrite was inhibitory at concentrations above 5 mM and had to be added in 2 portions (Figure 4). Moreover, adding 5 mM nitrate to sulfur-disproportionating culture did not influence the growth yield and amount of products of respiration. In comparison, 5 mM nitrite completely inhibited the growth with sulfur.

**Figure 5:**
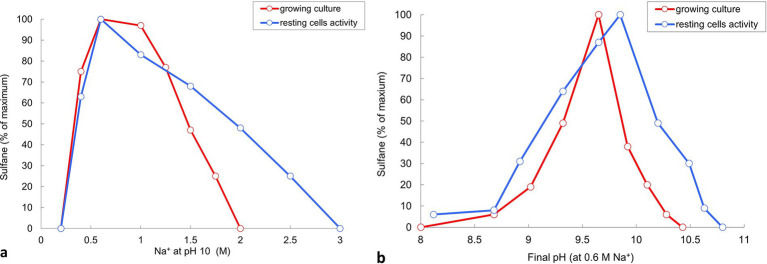
Influence of Na^+^ (carbonate buffer, pH 10) and pH (at 0.6 M total Na^+^) on sulfidogenic activity of strain AMeS2 at sulfur-disproportionating conditions in growing culture and resting cells. Sulfidogenic activity is shown as amount of total sulfanes formed by growing cultures (in red) or the grown cultures (resting cells, in blue). Results of duplicate experiments.

The influence of salinity in sodium carbonates and pH at 0.6 M total Na^+^ on sulfur disproportionation in AMeS2 was investigated both in growing cultures and on the level of catabolic activity of resting cells. Both ways showed the optimal concentration of 0.5- to 1 M total Na^+^ and optimal pH from 9.5–10. However, resting cells showed broader activity ranges for both salinity and pH compared to growing cultures (Figure 5). Overall, AMeS2 falls into the definition of a Cl-independent, moderately salt-tolerant obligate alkaliphile.

### General proteome properties of cells grown in the presence of different energy sources

**Figure 6:**
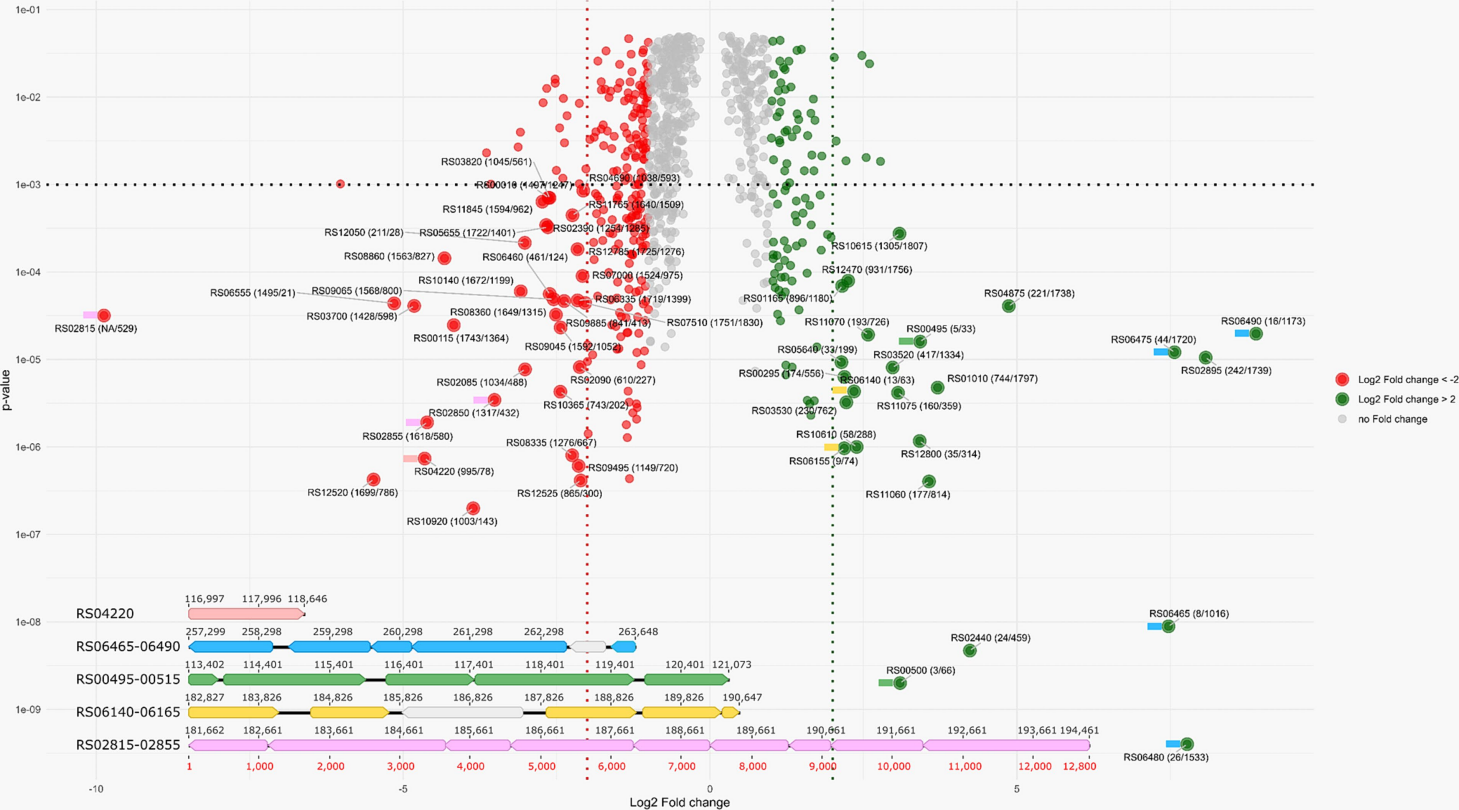
VulcanoPlot showing the AMeS2 gene expression during the growth by sulfur disproportionation and nitrate reduction with formate (DNRA). Positive fold change values indicate that the genes are upregulated during sulfur disproportionation, while negative values indicate that the genes are upregulated during DNRA. Horizontal dashed line indicates *p* = 0.001. Red and green dashed lines indicate log(2) fold change difference between two experiments equal to 2/−2 (i.e., the fold change = 4/−4). Green circles: upregulated (their expression level is above 2) during S^0^ disproportionation genes, red circles: upregulated (their expression level is above 2) at formate oxidation by nitrate genes. Large circles: the genes with *p* < 0.001 and the fold changes above 4 or below −4. The numbers in the circles are in the following format: the locus tag (protein rank by riBAQ calculated for S^0^ disproportionation culture/protein rank by riBAQ calculated for formate plus nitrate culture). Colored flags to the left of the proteins indicate that the genes encoding these proteins are part of the gene cluster of the same color, shown below the Volcano plot. Five gene clusters with highly regulated genes encoding the proteins playing significant roles in sulfur disproportionation or DNRA are shown. Pink: octaheme *c* nitrite reductase; blue: Psr; green: AprAB—QmoABC; yellow: Sat—DsrAB; light violet: octaheme *c* nitrite reductase. The genes of the proteins presumably not part of these enzyme complexes are in gray. The details are given in the main text.

Overall, 75% (1,864 of 2,491) of *in silico* predicted proteins were identified using proteomics across all experiments (Supplementary Table S1). A total of 127 genes were upregulated (their expression level was more than twice as high) and 268 genes were downregulated (their expression level was less than twice as low) during AMeS2 growth by S^0^ disproportionation compared to DNRA on formate (Figure 6; Supplementary Table S1).

### Mechanism of energy conservation revealed by genome and proteome analysis

#### Sulfur metabolism

**Figure 7:**
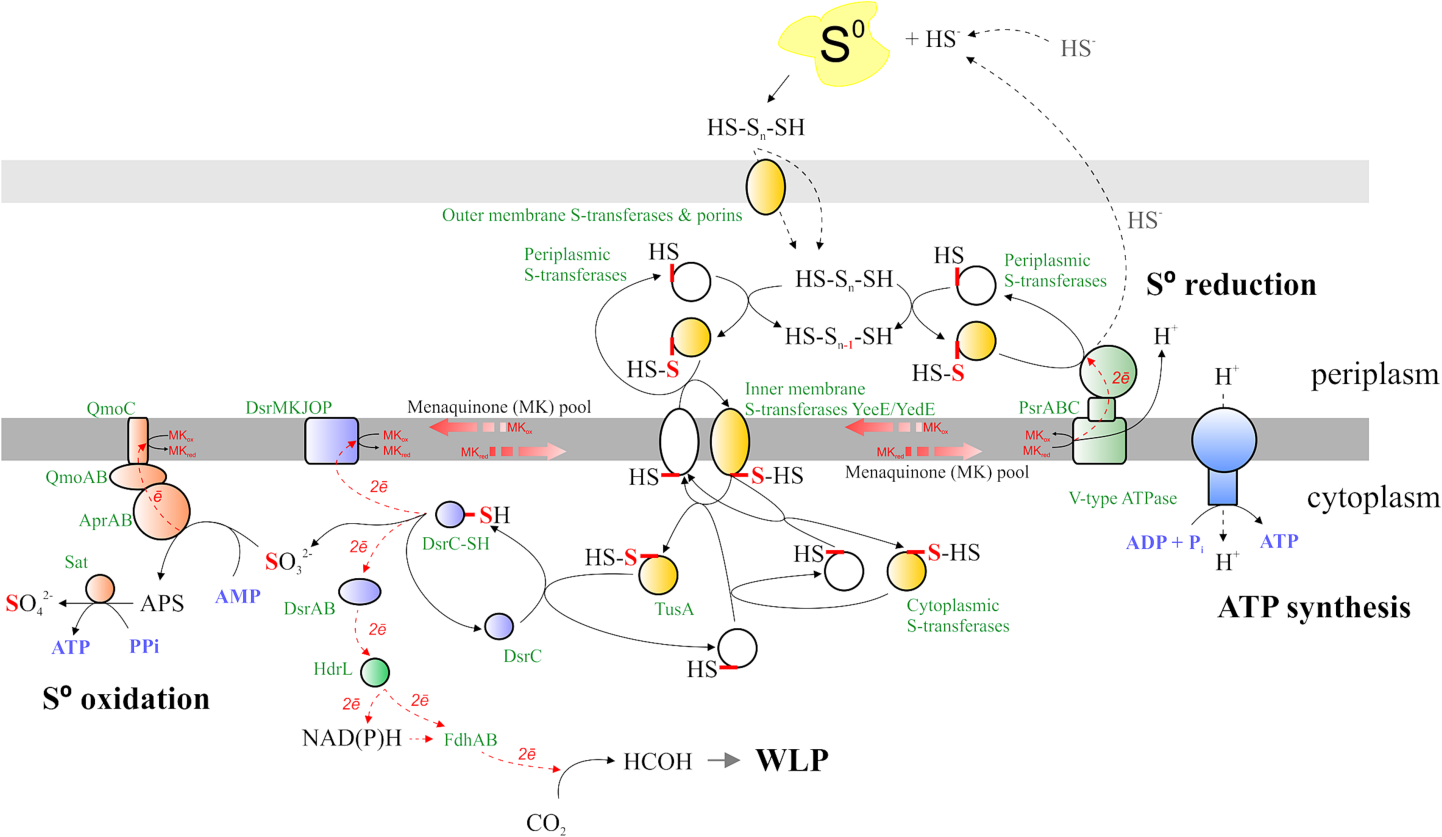
Sulfur metabolism of AMeS2. The mechanisms of S^0^ transfer, oxidation, and reduction, the possible connection of S^0^ oxidation and the WLP, and the spots and the sites of ATP synthesis are shown. Protein designations are shown in green; substrates and products are displayed in black; and nucleotide-phosphates and pyrophosphate are depicted in purple. The reduced/oxidized sulfur atom is in red. The electron flux is in the red dotted line. Qmo/Sat proteins are represented by orange shapes, Psr—green shapes, Dsr—purple shapes, ATP synthase—blue shapes, and sulfur transferases—white, while persulfurated sulfur transferases are in yellow. The details are given in the main text.

Sulfur reduction is catalyzed by polysulfide reductase (Psr, RS06465-06490), whose gene expression is strongly upregulated during AMeS2 growth by sulfur disproportionation (Figure 6). The *Psr* operon includes a gene (*RS06465*) encoding a sulfur-transferase with three rhodanese-like (PF00581, Pfam designation) domains. This sulfur-transferase has a signal peptide sequence indicating it accepts a sulfur atom from the sulfur donor outside the cytoplasmic membrane. Other secreted sulfur-transferases RS12800, RS05165, RS05685, and RS10230 are assumed to have a similar role. RS06465, RS12800, and RS05165 are predicted to be lipoproteins, implying they are most probably attached to the inner or outer membranes or both (Juncker et al., 2003), which might help them to interact with the Psr or extracellular polysulfide or to facilitate its import through the outer membrane. The import through the outer membrane might also involve porins, such as RS05170, whose gene is co-localized with the periplasmic sulfur-transferases RS12800, RS05165, and the inner-membrane sulfur-transferase YeeE/YedE (Figure 7). Another catalytic subunit, PsrA (RS10375), is encoded in the genome; however, there are no genes of other Psr subunits in the vicinity of *RS10375*, and its expression was relatively low in all experiments, which makes it unlikely that this protein is involved in sulfur reduction.

Sulfur-reducing and sulfur-oxidizing protein complexes are bound to or associated with membranes and exchange electrons through the menaquinones. While Psr acts in the periplasm, sulfur oxidation occurs in the cytoplasm, implying the need for the concerted action of periplasmic, inner membrane-bound, and cytoplasmic sulfur-transferases. RS12495, RS12500, RS05145, and RS10965 are cytoplasmic membrane sulfur-transferases YeeE/YedE, acquiring a sulfur atom from periplasmic polysulfide or periplasmic sulfur-transferases and donating it to cytoplasmic sulfur-transferases RS11585, RS12485, or directly to the TusA sulfur carrier (Figure 7). Among them, only RS12495 was shown to be overexpressed during disproportionation, yet the level of its expression during DNRA growth was also high (Supplementary Table S1). RS12500 and RS05145 were not detected during proteomic analysis, while RS10965 was downregulated during growth with sulfur, implying its possible action at low S concentrations.

Cytoplasmic membrane or cytoplasmic sulfur transferases transfer a sulfur atom to the cytoplasmic sulfur-carrier TusA (RS08955 and RS10930), which is known to interact with Dsr. Both *tusA* genes are not regulated under the tested conditions, while *RS10930* is one of the most expressed genes across all experiments, implying its constitutive expression (Supplementary Table S1). TusA further transfers the sulfur atom to the oxidative branch, which is a reversed sulfate reduction (rDsr, Dahl, 2017). No *DsrL* or *DsrEFH* genes, which are involved in sulfur oxidation in bacteria using the rDsr pathway (Stockdreher et al., 2014), were found in the AMeS2 genome, implying that, most probably, TusA itself persulfurates DsrC (RS03060). Resulting DsrC trisulfide is oxidized to DsrC and sulfite, providing two electrons to menaquinone via DsrMKJOP (RS12175-12195) and two electrons to DsrAB (RS06155-6160), which reduces an unknown acceptor (Figure 7)—an opposite reaction, proposed by Santos et al. (2015). The *dsrABCKO* are among the most expressed genes in all tested growth experiments. *dsrAB* are upregulated during sulfur disproportionation, while the expression of *dsrCMKJOP* genes appears to be constitutive (Supplementary Table S1).

Sulfite produced by the action of DsrC is further oxidized to sulfate via adenosine 5′-phosphosulfate (APS). These reactions are catalyzed by adenylylsulfate reductase AprAB (RS00500-00495), which donates electrons to menaquinones via a quinone-interacting membrane-bound oxidoreductase complex QmoABC (RS00505-00515), and by sulfate adenylyltransferase Sat (RS06140, Figure 7). All the genes of these proteins are among the most expressed in both experiments, and all of them except *qmoC* are upregulated during growth by sulfur disproportionation (Figure 6; Supplementary Table S1).

#### Formate metabolism

The AMeS2 genome encodes three formate dehydrogenases, Fdh/Fdn. The first one is a multi-subunit membrane-bound menaquinone-interacting FdnGHI (RS00990-00980), while the other two (RS03135 and RS08735) are cytoplasmic NAD(P)-dependent single proteins. Membrane-bound formate dehydrogenase is involved in periplasmic formate oxidation to CO_2_; electrons that are released in this process reduce menaquinones, which are further used by polysulfide reductase or nitrate and nitrite reductases during sulfur or nitrate/nitrite reduction, respectively. The catalytic *RS00990* is among the most highly expressed AMeS2 genes in all tested conditions and is slightly upregulated in the presence of formate (Figure 6; Supplementary Table S1).

Cytoplasmic Fdh RS08735 is a fused FdhAB protein, that is, it contains both a molybdopterin Fdh domain and NAD(P)H/FAD-binding and ferredoxin domains, implying it might be involved in the first step of the WLP methyl branch, catalyzing NAD(P)H-dependent CO_2_ reduction to formate. Two of its neighboring genes encode HdrA (RS08730) and MvhD (RS08725). HdrA is a FAD-containing heterodisulfide reductase, transferring electrons from ferredoxin to CoM-S-S-CoB. Still, it is also capable of electron bifurcation when the electrons from reduced coenzyme F420 are transferred to both ferredoxin and CoB-CoM heterodisulfide (Yan et al., 2017). HdrA of AMeS2 has two NADH/FAD-binding domains that make it resemble the HdrL of *Desulfobacterium autotrophicum*—an autotrophic marine sulfate-reducing bacterium (Strittmatter et al., 2009). Three HdrL proteins of *Db. autotrophicum* and MvhD domain-containing proteins are proposed to be involved in electron cycling during sulfidogenesis (Strittmatter et al., 2009). Here, we suggest a similar role but in sulfur oxidation and in connecting sulfur and carbon metabolism. We hypothesize that HdrA/HdrL (RS08730) can accept electrons provided by DsrC trisulfide through DsrAB and shuttle them directly or via NAD(P)H to the FdhAB (RS08735) for the first step of CO_2_ reduction in the methyl branch of WLP (Figure 7). In addition to the co-localisation of the *fdhAB*, *hdrL*, and *mvhD* (*RS08735-08725*) together with the genes of the WLP (*RS08760-08740*), the proposed interaction of HdrL and FdhAB is evidenced by similar expression of the genes of the whole cluster *RS08760-08725* (Supplementary Table S1).

In contrast to the two other Fdh, the gene of the second cytoplasmic FdhA—*RS03135*—is not among the highly expressed genes, but it is upregulated during sulfur disproportionation. Two of its neighbors encode NAD(P)H oxidizing HydB (RS03130) and HydG (RS03125) subunits of the cytoplasmic sulfhydrogenase. Since subunits HydA (NiFe hydrogenase) and HydD are absent, it can be hypothesized that in AMeS2, instead of transferring electrons from NAD(P)H to HydA and hydrogen, HydB and HydG may shuttle them to the FdhA (RS03135) for the reduction of CO_2_ to formate. Therefore, the FdhA-HydA-HydD complex might be an additional starting point of the methyl branch of the WLP; however, taking into account the low expression of *RS03125-03135* genes, their role in autotrophic metabolism of AMeS2 is likely to be minor, at least at the tested conditions.

#### Nitrate metabolism

During AMeS2 growth on formate and nitrate, the first is oxidized by the membrane-bound FdnGHI (RS00990-00980), while the reduction of nitrate to ammonium is assumed to be due to several enzyme complexes. Nitrate reduction to nitrite catalyzed by a periplasmic nitrate reductase of the Nap family (NapAGHD RS11645-11630, Simon and Klotz, 2013). Proteomic analysis revealed that the NapA is strongly upregulated during growth on nitrate, as are the genes for another representative of molybdopterin oxidoreductases (RS05665-05655). Phylogenetic analysis of catalytic subunits (Supplementary Figure S3) supported affiliation of RS11645 with the Nap clade. However, the second catalytic subunit RS05660 fell within the arsenate reductases family Arr. At the same time, proteomic analysis suggests both are involved in nitrate respiration. In AMeS2 the genes of Arr subunits located in the order *ArrCAB* while the canonical Arr subunit genes context is *arrACB* (Duval et al., 2008), which, together with the proteomics results, indicates that the activity of this enzymatic complex is different from arsenate reductase and, in particular, related to the reduction of nitrate. Two nearby genes (*RS05675-05670*) encode proteins involved in the regulation of nitrate/nitrite reduction, making this assumption more plausible.

Nitrite reduction to ammonium might be catalyzed by two periplasmic octaheme *c* proteins: RS02815-02855 and RS04220. None resembles NrfAH—a well-studied DNRA enzyme with a pentaheme *c* catalytic subunit. RS04220 contains a lysine-containing heme *c* (CXXC**K**) shown to be present in ammonifying nitrite reductases (Besson et al., 2022). This protein is homologous (44% identity, 92% coverage) to the biochemically characterized enzyme TaNiR from *T. ammonificans* with a proven *in vivo* function as an ammonifying nitrite reductase (WP_305732658, Sorokin et al., 2023). The second octaheme *c* RS02855 is a part of the genomic locus (*RS02815-02855*) coding for several extracellular multiheme *c* proteins, and might have an altogether different function. However, similar to *RS04220*, the genes of this cluster are highly upregulated (and *RS02855* is the most upregulated gene) in DNRA conditions (Figure 6; Supplementary Table S1), indicating that this complex might play the role of an alternative nitrite reductase.

The genome of AMeS2 contains a large gene cluster encoding nitrogenase NifDHK and auxiliary proteins NifABENO (RS03865-03900). However, attempts to grow AMeS2 in nitrogen-free medium were not successful.

Complex I (Nuo) provides additional energy conservation during anaerobic respiration. In AMeS2, the genes for subunits of the exporting proton membrane arm (NuoAHJKLMN) and reducing quinones Q-module (NuoBCDI, Kampjut and Sazanov, 2022) are present in one cluster (*RS03515-03575*); however, the N-module (NuoEFG), essential for NADH reduction, appears to be absent. NuoEFG homologs (RS00815-00805) are present, but they most probably represent NADH-subunits of cytoplasmic enzyme complexes such as hydrogenases Hox or Mvh, since the homologous subunits of these enzymes are among their nearest biochemically characterized relatives, and *RS00815-00805* genes located closely to other hydrogenase subunits (*RS00800-00795*) and distantly to the Nuo gene cluster. The Nuo-like genes are not among the most expressed genes in AMeS2, but they are strongly upregulated during sulfur disproportionation, suggesting that an additional energy conservation mechanism is important for this thermodynamically challenging catabolic process. The existing Nuo proteins may interact with some unknown electron-donating oxidoreductase, or the NADH-dehydrogenase subunits, predicted to be part of Hox/Mvh, indeed represent NuoEFG.

Sulfide-quinone reductase Sqr (RS02270) may also be involved in reducing menaquinones by electrons from sulfide oxidation to polysulfide. Although the protein lacks detectable signal peptide and TM helices, it is predicted to be secreted via an unknown mechanism. On the other hand, its gene is not well expressed and was not upregulated in the presence of sulfur, making it difficult to expect that it has a vital role in sulfur catabolism.

The genes encoding subunits of hydrogen-translocating F-type ATP-synthase (RS11675-11705 and RS04835-04850) are among the most expressed in all tested conditions.

## Conclusion

Isolated initially as a sulfur disproportionating microorganism *D. dismutans* strain AMeS2 is a novel species of the genus *Desulfurivibrio* and only the second characterized haloalkaliphilic bacterium capable of sulfur disproportionation. This energy-limited anaerobic metabolism relies on sulfur respiration, catalyzed by the periplasmic membrane-bound polysulfide reductase Psr and reversed sulfate reduction, catalyzed by the intracellular Dsr complex. AMeS2 is a relatively highly specialized bacterium, capable of, in addition to S^0^ disproportionation, growing by formate-dependent sulfur reduction to sulfide or nitrate reduction to ammonium (DNRA). Like most known sulfur disproportionators, AMeS2 is an obligate autotroph, using the Wood–Ljungdahl pathway (WLP) of CO_2_ assimilation. While one membrane-bound formate dehydrogenase, FdnGHI, is involved in the oxidation of formate, either by sulfur- or nitrate-dependent respiratory processes, two other formate dehydrogenases are supposed to be involved in cytoplasmic CO_2_ reduction to formate—the first step of the methyl branch of the WLP. A possible source of electrons for them could be DsrAB and HdrL, allowing us to suggest that these enzymes link the carbon and sulfur cycles by shuffling electrons from sulfur to CO_2_. This tight coupling between the sulfur and carbon cycles may be advantageous under the stringent energetic conditions of sulfur disproportionation in which this microorganism exists.

## Description of *D. dismutans* sp. nov.

dis.mu’tans Gr. adv. *Dis*, in two, apart; L. press. Part. *mutans*, changing, altering; N.L. part. Adj. *dismutans*, dismutating, splitting.

Cells of AMeS2 are short comma/vibrio-shaped, 0.4–0.5 μm in width and 1.5–2 μm in length, motile with 1–2 thick sublateral flagella. The colonies developing within a sulfur-containing soft agar are black and have the shape of lenses up to 1 mm in diameter. Strictly anaerobic obligate chemolithoautotroph with a respiratory metabolism. Utilizes elemental sulfur, nitrate, and nitrite as the electron acceptors and sulfur or formate as the electron donors. Grows by sulfur disproportionated into sulfide and sulfate (without addition of ferric iron) and by formate-dependent dissimilatory sulfur reduction to sulfide or nitrate/nitrite reduction to ammonium. Thiosulfate and sulfite are not disproportionated. H_2_, CO, acetate, pyruvate, succinate are not utilized without an electron acceptor or with sulfur, thiosulfate, sulfate, or nitrate as the electron acceptors. CO_2_ is fixed by the Wood–Lijundahl pathway. Obligately alkaliphilic, with a pH range for growth between 8.5 and 10.25 (an optimum at 9.5) and moderately salt-tolerant, with a [Na^+^] range for growth of 0.3–1.75 M (optimum at 0.5–1.0 M). Mesophilic, with a maximal temperature for growth at 37–40°C. The G + C content of the DNA is 60.0% (inferred through the genome sequence analysis). The GenBank accession number of the genome assembly is GCF_029210385. The type strain was isolated from the mixed anaerobic sediments of soda lakes in southwestern Siberia (Altai region, Russia). The type strain is AMeS2 (DSM 113758; JCM 39203; and UNIQEM U934).

## Data Availability

The datasets presented in this study can be found in online repositories. The names of the repository/repositories and accession number(s) can be found in the article/Supplementary material.

## Footnotes

^1^ http://ggdc.dsmz.de/ggdc.php
