# Supplementary material for: Growth physiology, genomics, and proteomics of Desulfurivibrio dismutans sp. nov., an obligately chemolithoautotrophic, sulfur disproportionating and ammonifying haloalkaliphile from soda lakes
Source: Front Microbiol. 2025 May 23;16:1590477. doi: 10.3389/fmicb.2025.1590477 (PMC12142623; doi:10.3389/fmicb.2025.1590477)
Supplement: Supplementary file 2 [file Data_Sheet_1.pdf]

Supplemental materials

Growth physiology, genomics and proteomics of *Desulfurivibrio dismutans* sp. nov., an obligately chemolithoautotrophic, sulfur-disproportionating and ammonifying haloalkaliphile from soda lakes

Dimitry Y. Sorokin[1,2*], Alexander Y. Merkel[1], Rustam H. Ziganshin[3], Ilya V. Kublanov[4*]

[1]*Winogradsky Institute of Microbiology, Federal Research Centre of Biotechnology, Russian Academy of Sciences, Moscow, Russia*

[2]*Department of Biotechnology, Delft University of Technology, Delft, The Netherlands*

[3]*Shemyakin-Ovchinnikov Institute of Bioorganic Chemistry, Russian Academy of Sciences, Moscow, Russia*

[4]*Department of Plant Pathology and Microbiology, The Robert H. Smith Faculty of Agriculture, Food and Environment, The Hebrew University of Jerusalem, Rehovot, Israel*

*Corresponding authors: Dimitry Y. Sorokin d.sorokin@tuadelft.nl; Ilya V. Kyblanov ilya.kublanov@mail.huji.ac.il

Table S1. Proteomic analysis of *Desulfurivibrio dismutans* strain AMeS2 grown by S° disproportionation compared to growth by DNRA on formate.

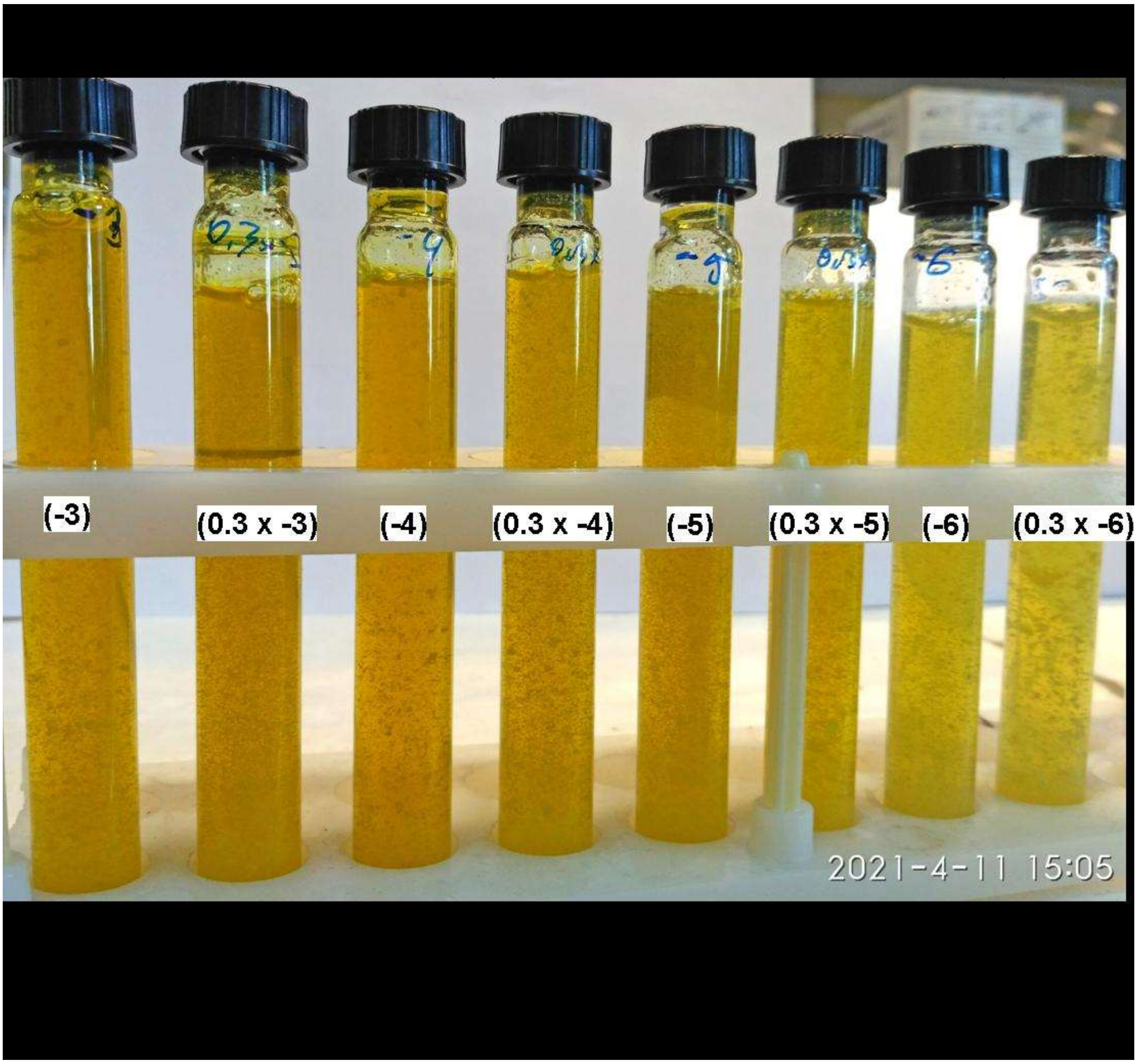

Figure S1. Shake-agar dilution series at sulfur-disproportionating conditions resulting in formation of single colonies from one of which *Desulfurivibrio* sp. strain AMeS2 was isolated. The medium has a pH 10 and contain 0.6 M total Na^+.

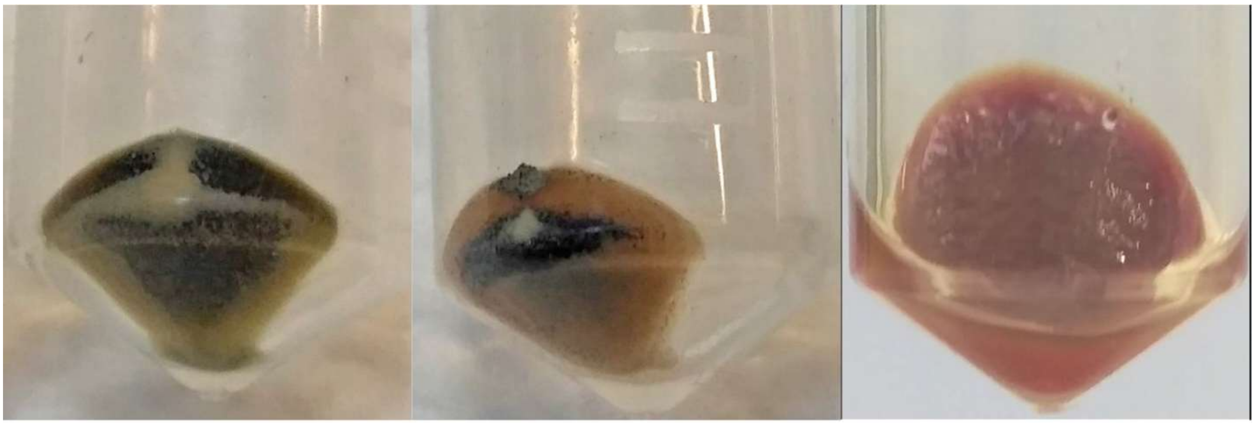

Figure S2. Difference in biomass color of strain AMeS2 grown by sulfur disproportionation (left), sulfur + formate (center) and formate + nitrate (right).

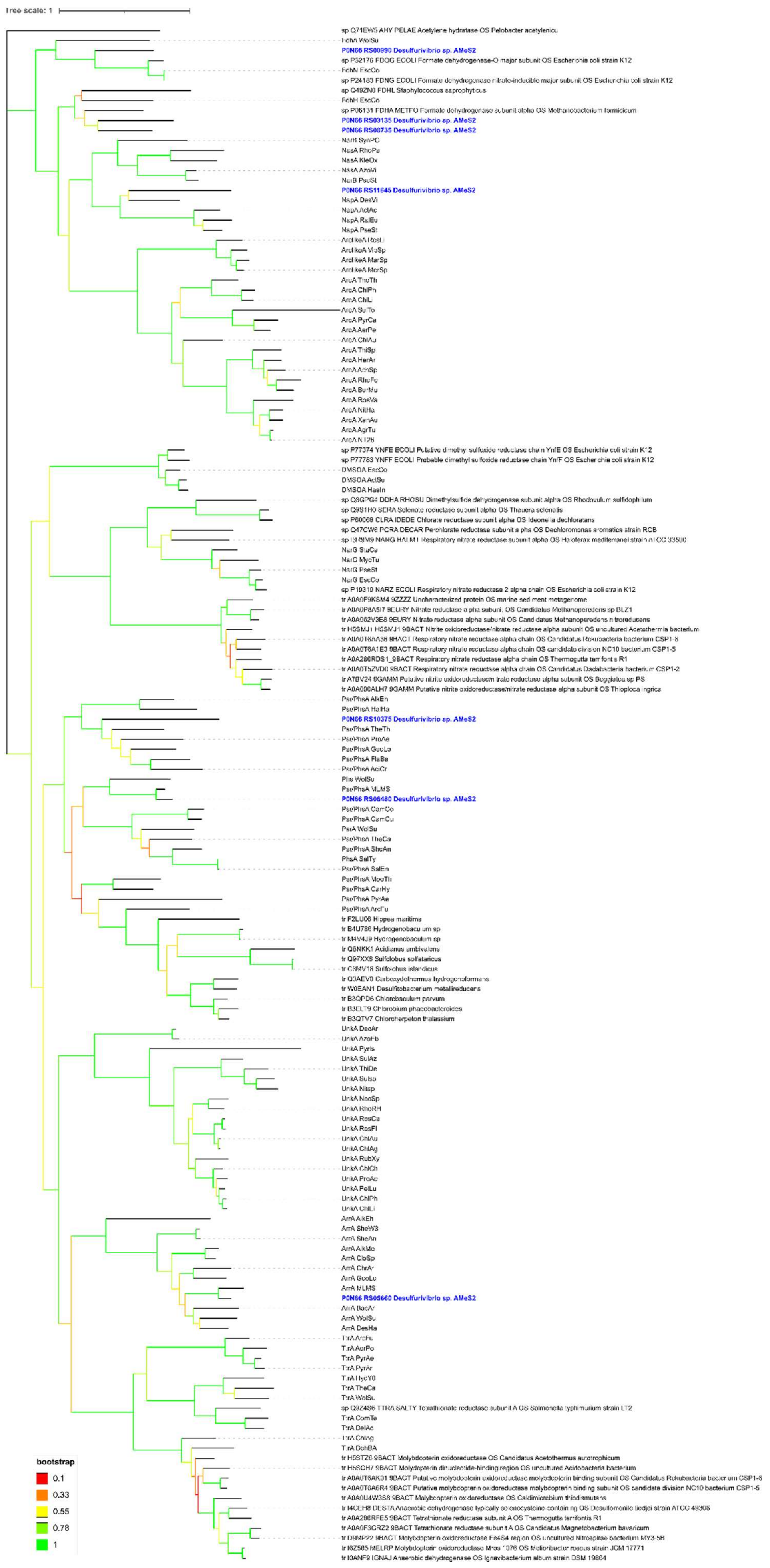
Tree scale: 1
sp Q71EW5 AHY PELAE Acetylene hydratase OS Pelobacter acetylenicu
FchA WolSu
P0N66 RS00990 Desulfurivibrio sp. AMeS2
sp P32176 FDOG ECOLI Formate dehydrogenase-O major subunit OS Escherichia coli strain K12
FchN EscCo
sp P24183 FDNG ECOLI Formate dehydrogenase nitrate-inducible major subunit OS Escherichia coli strain K12
sp Q49ZN0 FDHL Staphylococcus saprophyticus
FchH EscCo
sp P06131 FDHA METFO Formate dehydrogenase subunit alpha OS Methanobacterium formicicum
P0N66 RS03135 Desulfurivibrio sp. AMeS2
P0N66 RS08735 Desulfurivibrio sp. AMeS2
NarB SynPC
NasA RhoPa
NasA KleOx
NasA AzoVi
NarB PseSt
P0N66 RS11645 Desulfurivibrio sp. AMeS2
NapA DesVi
NapA ActAc
NapA RalEu
NapA PseSt
ArolikeA RosLi
ArolikeA VioSp
ArolikeA MarSp
ArolikeA MorSp
AroA TheTh
AroA ChlPh
AroA ChlLi
AroA SulTo
AroA PyrCa
AroA AerPe
AroA ChlAu
AroA ThiSp
AroA HerAr
AroA AchSp
AroA RhoFe
AroA BurMu
AroA RosVa
AroA NitHa
AroA XanAu
AroA AgrTu
AroA NT26
sp P77374 YNFE ECOLI Putative dimethyl sulfoxide reductase chain YnfE OS Escherichia coli strain K12
sp P77783 YNFF ECOLI Probable dimethyl sulfoxide reductase chain YnfF OS Escherichia coli strain K12
DMSOA EscCo
DMSOA ActSu
DMSOA HaeIn
sp Q8GPG4 DDHA RHOSU Dimethylsulfide dehydrogenase subunit alpha OS Rhodovulum sulfidophilum
sp Q9S1H0 SERA Selenate reductase subunit alpha OS Thauera selenatis
sp P60068 CLRA IDEDE Chlorate reductase subunit alpha OS Ideonella dechloratans
sp Q47CW6 PCRA DECAR Perchlorate reductase subunit alpha OS Dechloromonas aromatica strain RCB
sp I3R9M9 NARG HALMT Respiratory nitrate reductase subunit alpha OS Haloferax mediterranei strain ATCC 33500
NarG StaCa
NarG MycTu
NarG PseSt
NarG EscCo
sp P19319 NARZ ECOLI Respiratory nitrate reductase 2 alpha chain OS Escherichia coli strain K12
tr A0A0F9KSM4 9ZZZZ Uncharacterized protein OS marine sediment metagenome
tr A0A0P8A5I7 9EURY Nitrate reductase alpha subunit OS Candidatus Methanoperedens sp BLZ1
tr A0A062V3E8 9EURY Nitrate reductase alpha subunit OS Candidatus Methanoperedens nitroreducens
tr H5SMJ1 H5SMJ1 9BACT Nitrite oxidoreductase/nitrate reductase alpha subunit OS uncultured Acetothermia bacterium
tr A0A0T6AA36 9BACT Respiratory nitrate reductase alpha chain OS Candidatus Rokubacteria bacterium CSP1-6
tr A0A0T6A1E3 9BACT Respiratory nitrate reductase alpha chain OS candidate division NC10 bacterium CSP1-5
tr A0A286RDS1_9BACT Respiratory nitrate reductase alpha chain OS Thermogutta terrifontis R1
tr A0A0T5ZVD0 9BACT Respiratory nitrate reductase alpha chain OS Candidatus Dadabacteria bacterium CSP1-2
tr A7BV24 9GAMM Putative nitrite oxidoreductase/nitrate reductase alpha subunit OS Beggiatoa sp PS
tr A0A090ALH7 9GAMM Putative nitrite oxidoreductase/nitrate reductase alpha subunit OS Thioploca ingrica
Psr/PhsA AlkEh
Psr/PhsA HalHa
P0N66 RS10375 Desulfurivibrio sp. AMeS2
Psr/PhsA TheTh
Psr/PhsA ProAe
Psr/PhsA GeoLo
Psr/PhsA FlaBa
Psr/PhsA AciCr
Phs WolSu
Psr/PhsA MLMS
P0N66 RS06480 Desulfurivibrio sp. AMeS2
Psr/PhsA CamCo
Psr/PhsA CamCu
PsrA WolSu
Psr/PhsA TheCa
Psr/PhsA SheAn
PhsA SalTy
Psr/PhsA SalEn
Psr/PhsA MooTh
Psr/PhsA CarHy
Psr/PhsA PyrAe
Psr/PhsA ArcFu
tr F2LU06 Hippea maritima
tr B4U786 Hydrogenobaculum sp
tr M4V4J9 Hydrogenobaculum sp
tr Q6NKK1 Acidianus ambivalens
tr Q97XX8 Sulfolobus solfataricus
tr C3MV18 Sulfolobus islandicus
tr Q3AEV0 Carboxydothermus hydrogenoformans
tr W0EAN1 Desulfitobacterium metallireducens
tr B3QPD6 Chlorobaculum parvum
tr B3ELT9 Chlorobium phaeobacteroides
tr B3QTV7 Chloroherpeton thalassium
UnkA DecAr
UnkA AzoEb
UnkA PyrIs
UnkA SulAz
UnkA ThiDe
UnkA SulsP
UnkA Nitsp
UnkA NaoSp
UnkA RhoRH
UnkA RosCa
UnkA RosFl
UnkA ChlAu
UnkA ChlAg
UnkA RubXy
UnkA ChlCh
UnkA ProAe
UnkA PelLu
UnkA ChlPh
UnkA ChlLi
ArrA AlkEh
ArrA SheW3
ArrA SheAn
ArrA AlkMo
ArrA CloSp
ArrA ChrAr
ArrA GeoLo
ArrA MLMS
P0N66 RS05660 Desulfurivibrio sp. AMeS2
ArrA BacAr
ArrA WolSu
ArrA DesHa
TtrA ArcFu
TtrA AerPe
TtrA PyrAe
TtrA PyrAr
TtrA HycY0
TtrA TheCa
TtrA WolSu
sp Q9Z4S6 TTRA SALTY Tetrathionate reductase subunit A OS Salmonella typhimurium strain LT2
TtrA ComTe
TtrA DelAc
TtrA Chlag
TtrA DehBA
tr H5STZ6 9BACT Molybdopterin oxidoreductase OS Candidatus Acetothermus autotrophicum
tr H5SCH7 9BACT Molydopterin dinucleotide-binding region OS uncultured Acidobacteria bacterium
tr A0A0T6AK31 9BACT Putative molybdopterin oxidoreductase molybdopterin binding subunit OS Candidatus Rokubacteria bacterium CSP1-6
tr A0A0T6A6R4 9BACT Putative molybdopterin oxidoreductase molybdopterin binding subunit OS candidate division NC10 bacterium CSP1-5
tr A0A0U4W3S8 9BACT Molybdopterin oxidoreductase OS Caldimicrobium thiodismutans
tr I4CEH8 DESTA Anaerobic dehydrogenase typically selenocysteine-containing OS Desulfomonile tiedjei strain ATCC 49306
tr A0A286RFE5 9BACT Tetrathionate reductase subunit A OS Thermogutta terrifontis R1
tr A0A0F3GRZ2 9BACT Tetrathionate reductase subunit A OS Candidatus Magnetobacterium bavaricum
tr D9MP22 9BACT Molybdopterin oxidoreductase Fe4S4 region OS uncultured Nitrospirae bacterium MY3-5B
tr I6Z5E5 MELRP Molybdopterin oxidoreductase Mros 1076 OS Melioribacter roseus strain JCM 17771
tr I0ANF9 IGNAJ Anaerobic dehydrogenase OS Ignavibacterium album strain DSM 19864
bootstrap
0.1
0.33
0.55
0.78
1

Figure S3. Phylogenetic analysis of catalytic subunits of the molybdopterin oxidoreductases. The tree was constructed in MEGA X (Kumar et al., 2018) using the Maximum Likelihood method and Le_Gascuel_2008 model (Le and Gacuel, 2008). The tree with the highest log likelihood (-110986.93) is shown. The percentages of trees in which the associated taxa clustered together (bootstrap values) are shown as heat lines. The tree is drawn to scale, with branch lengths measured in the number of substitutions per site. This analysis involved 154 amino acid sequences. All positions with less than 95% site coverage were eliminated. There were a total of 535 positions in the final dataset. Sequence designations are as follows: sequences with the short designations were taken from the dataset of the molybdopterin oxidoreductases made by Duval et al., 2008. Sequences with long designations, started with "tr" or "sp" representing the nearest relatives of the AMeS2 molybdopterin oxidoreductases were downloaded from the Uniprot/Swissprot. AMeS2 molybdopterin oxidoreductases are in bold blue. The tree was beautified in iTOL (Letunic and Bork, 2024) and Inkscape v 0.91.

- Duval S, Ducluzeau AL, Nitschke W, Schoepp-Cothenet B. Enzyme phylogenies as markers for the oxidation state of the environment: the case of respiratory arsenate reductase and related enzymes. *BMC Evol Biol.* 2008; 8:206.
- Kumar S., Stecher G., Li M., Knyaz C., and Tamura K. MEGA X: Molecular Evolutionary Genetics Analysis across computing platforms. *Molecular Biology and Evolution* 2018; 35:1547-1549.
- Le S.Q. and Gascuel O. An Improved General Amino Acid Replacement Matrix. *Mol Biol Evol* 2008; 25(7):1307-1320.
- Letunic I and Bork P. Interactive Tree of Life (iTOL) v6: recent updates to the phylogenetic tree display and annotation tool. *Nucleic Acids Res* 2024 52(W1):W78–W82.
